# Downregulation of cyclin D1 sensitizes cancer cells to MDM2 antagonist Nutlin-3

**DOI:** 10.18632/oncotarget.8999

**Published:** 2016-04-26

**Authors:** Peipei Yang, Weicai Chen, Xuhui Li, Grant Eilers, Quan He, Lili Liu, Yeqing Wu, Yuehong Wu, Wei Yu, Jonathan A. Fletcher, Wen-Bin Ou

**Affiliations:** ^1^ Zhejiang Provincial Key Laboratory of Silkworm Bioreactor and Biomedicine, College of Life Sciences, Zhejiang Sci-Tech University, Hangzhou, China; ^2^ Zhejiang Provincial Key Laboratory of Applied Enzymology, Yangtze Delta Region Institute of Tsinghua University, Jiaxing, Zhejiang, China; ^3^ Department of Pathology, Brigham and Women's Hospital and Harvard Medical School, Boston, MA, USA

**Keywords:** cyclin D1, MDM2, p53, upregulation, Nutlin-3

## Abstract

The MDM2-p53 pathway has a prominent oncogenic function in the pathogenesis of various cancers. Nutlin-3, a small-molecule antagonist of MDM2-p53 interaction, inhibits proliferation in cancer cells with wild-type p53. Herein, we evaluate the expression of MDM2, both the full length and a splicing variant MDM2-A, and the sensitivity of Nutlin-3 in different cancer cell lines. Included are seven cell lines with wild-type p53 (four mesothelioma, one breast cancer, one chondrosarcoma, and one leiomyosarcoma), two liposarcoma cell lines harboring MDM2 amplification and wild-type p53, and one mesothelioma cell line harboring a p53 point mutation. Nutlin-3 treatment increased expression of cyclin D1, MDM2, and p53 in cell lines with wild-type p53. Additive effects were observed in cells containing wild-type p53 through coordinated attack on MDM2-p53 binding and cyclin D1 by lentivirual shRNA knockdown or small molecule inhibition, as demonstrated by immunoblots and cell viability analyses. Further results demonstrate that MDM2 binds to cyclin D1, and that an increase in cyclin D1 expression after Nutlin-3 treatment is correlated with expression and ubiquitin E3-ligase activity of MDM2. MDM2 and p53 knockdown experiments demonstrated inhibition of cyclin D1 by MDM2 but not p53. These results indicate that combination inhibition of cyclin D1 and MDM2-p53 binding warrants clinical evaluation as a novel therapeutic strategy in cancer cells harboring wild-type p53.

## INTRODUCTION

The human homolog of mouse double minute 2 (MDM2) is an oncoprotein overexpressed in different types of malignant cancers, due to gene amplification or the SNP309 polymorphism in the promoter region of *MDM2* [1, 2]. MDM2 E3 ubiquitin ligase activity is responsible for the ubiquitination and degradation of the tumor suppressor p53 [3]. The structure of MDM2 bound to p53 and the molecular mechanisms of the interaction between MDM2 and p53 have been well characterized [4–6]. MDM2 binds p53 at its N-terminal transactivation domain, blocking p53 transcriptional function, while simultaneously, through the C-terminal RING-finger domain, promoting its polyubiquitination and proteasome-dependent degradation [7]. p53 enhances *MDM2* transcription through p53-specific response elements in the promoter region of *MDM2*, thus forming an autoregulatory feedback loop critical to controlling the balance of p53 and MDM2 [8]. Nutlin-3, a specific small-molecule inhibitor of MDM2, blocks the binding of MDM2 with wild type p53, activating the anticancer activity of p53 [9–11]. Previous reports have confirmed that Nutlin-3 inhibits cell viability in cancer cell lines harboring wild-type p53, with an attenuated effect in cell lines harboring mutant p53, indicating that p53 mutation is a strong negative predictor of response [11, 12]. Additionally, many wild-type p53 cell lines have shown varying sensitivities to Nutlin-3 [12].

Many MDM2 splicing variants have been described in a variety of malignancies, including ovarian cancer [13], breast cancer [14], non-small cell lung cancer [15], and liposarcoma [16], revealing the complexity of the MDM2 and p53 feedback loop. Alternative MDM2 transcripts mainly include MDM2-FL (full length, ~90kDa), MDM2-A (~60 kDa), MDM2-B (~45 kDa), MDM2-C, MDM2-HL1, and MDM2-HL2 (~30 kDa) [17]. A number of variant transcripts may not retain the entire p53 binding site, therefore limiting the response of cancer cells to Nutlin-3 [16, 17]. MDM2 splicing variants are tumorigenic in mouse models [14] and have been associated with tumor progression in breast carcinomas. However, the biological function of aberrantly spliced MDM2 isoforms varies from tumor promotion to growth arrest [13, 18]. For example, MDM2-B stimulates the growth of mouse embryo fibroblasts and exerts antiapoptotic effects [19]. Notably, expression of MDM2-A and MDM2-B increases the expression levels of cyclin D1 [17], which has an oncogenic role in cancer cells.

In this study, we seek to understand the different responses to the MDM2 inhibitor Nutlin-3 in wild-type p53 cancer cells and the determinants of Nutlin-3 sensitivity and/or resistance. We evaluate the expression of MDM2-FL, MDM2-A and cyclin D1 in different cancer cell lines. We examine the effects of MDM2 and cyclin D1 inhibition on proliferation and survival and explore the regulation mechanisms of cyclin D1 by MDM2. These studies suggest that combination inhibition of MDM2 and cyclin D1 warrants clinical evaluation as a therapeutic strategy in wild-type p53 cancers.

## RESULTS

### Expression of MDM2 in cancer cell lines

**Figure 1:**
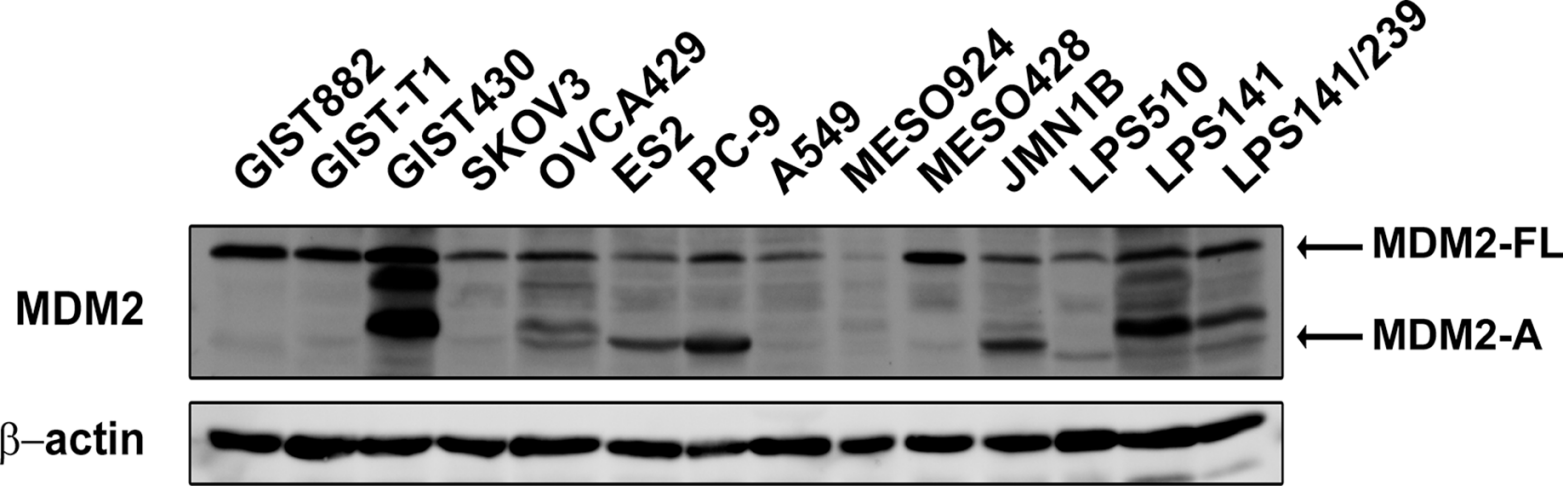
Expression of MDM2 in cancer cell lines Immunoblotting evaluation of MDM2-FL and MDM2-A expression in cancer cell lines including gastrointestinal stromal tumor (GIST882, GIST-T1, and GIST430), ovarian cancer (SKOV3, OVCA429, and ES2), non-small-cell lung cancer (PC-9 and A549), mesothelioma (MESO924, MESO428, and JMN1B), and liposarcoma (LPS510, LPS141, and LPS141/239). Actin staining is a loading control.

Expression of MDM2 full length (FL) and alternatively spliced variant MDM2-A was evaluated by immunoblotting in a group of cancer cell lines including GIST cell lines (GIST882, GIST-T1, and GIST430), breast cancer cell lines (SKOV3, OVCA429, and ES2), non-small cell lung cancer cell lines (PC-9 and A549), mesothelioma cell lines (MESO924, MESO428, and JMN1B), and liposarcoma cell lines (LPS510, LPS141, and LPS141/239) (Figure 1). The complete transcript (MDM2-FL) was detected in all cell lines, and the shorter isoform, MDM2-A, was detected in most cell lines, regardless of the mutational status of p53 and the amplification level of *MDM2* (Figure 1).

### Induction of cyclin D1 by Nutlin-3 treatment

**Figure 2:**
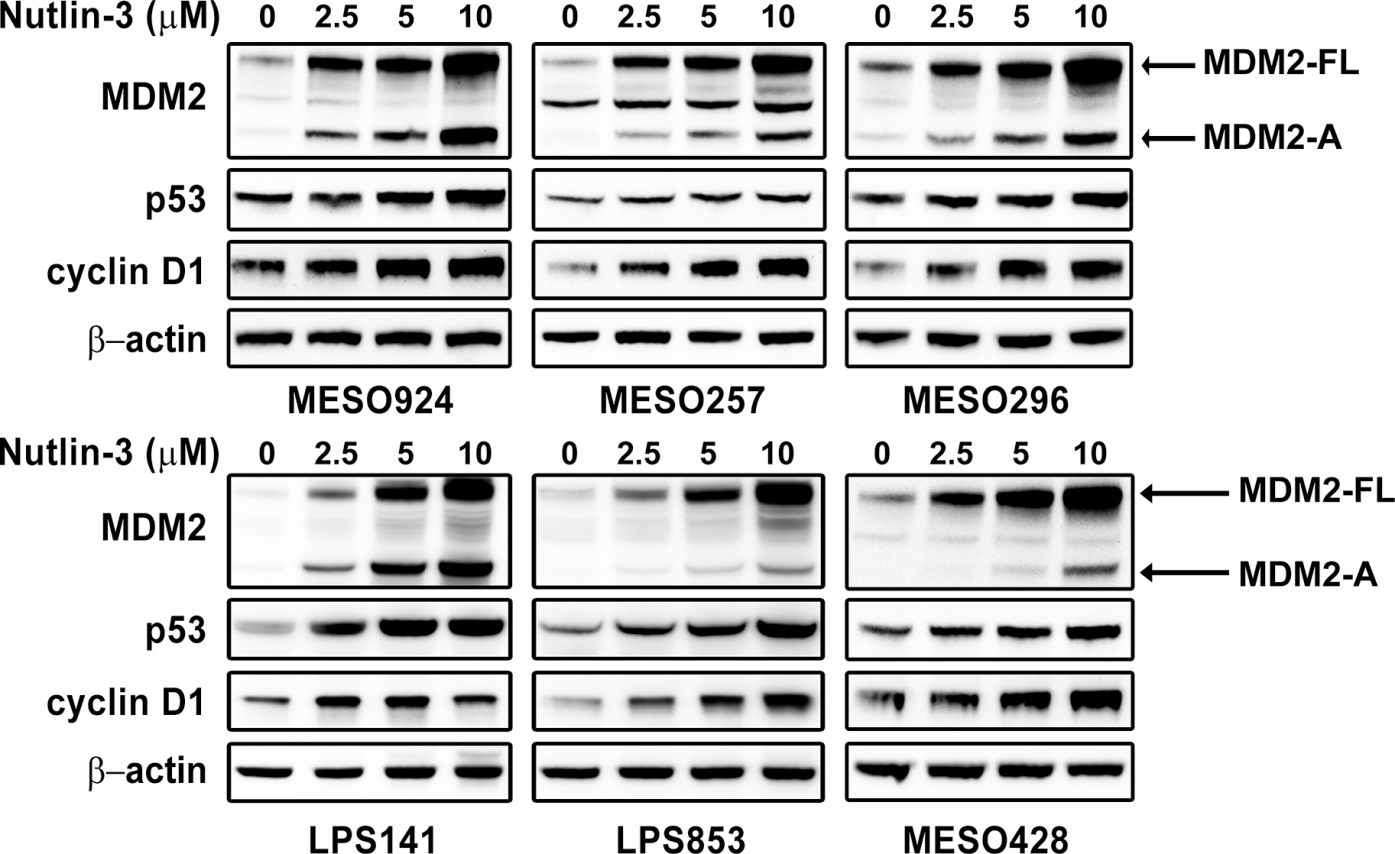
Immunoblotting evaluation of the effects of MDM2 inhibitor Nutlin-3 (2.5, 5, 10 μM) on expression of MDM2, p53, and cyclin D1 in mesothelioma (MESO924, MESO257, MESO296, and MESO428) and liposarcoma (LPS141 and LPS853) total cell lysates after 48 hours of treatment in serum-containing medium β-actin stain is a loading control.

Expression of MDM2, p53, and cyclin D1 was evaluated by immunoblotting in mesothelioma and LPS cell lines after Nutlin-3 treatment for 48 hours (Figure 2). Nutlin-3 treatment induced expression of MDM2-FL, MDM2-A and p53 in these cell lines in a dose-dependent manner (Figure 2). In addition, treatment with Nutlin-3 resulted in 2-3-fold increase in cyclin D1 expression compared to the DMSO control treatment (Figure 2).

### Additive effects of simultaneous inhibition of MDM2-p53 interaction and cyclin D1

**Figure 3:**
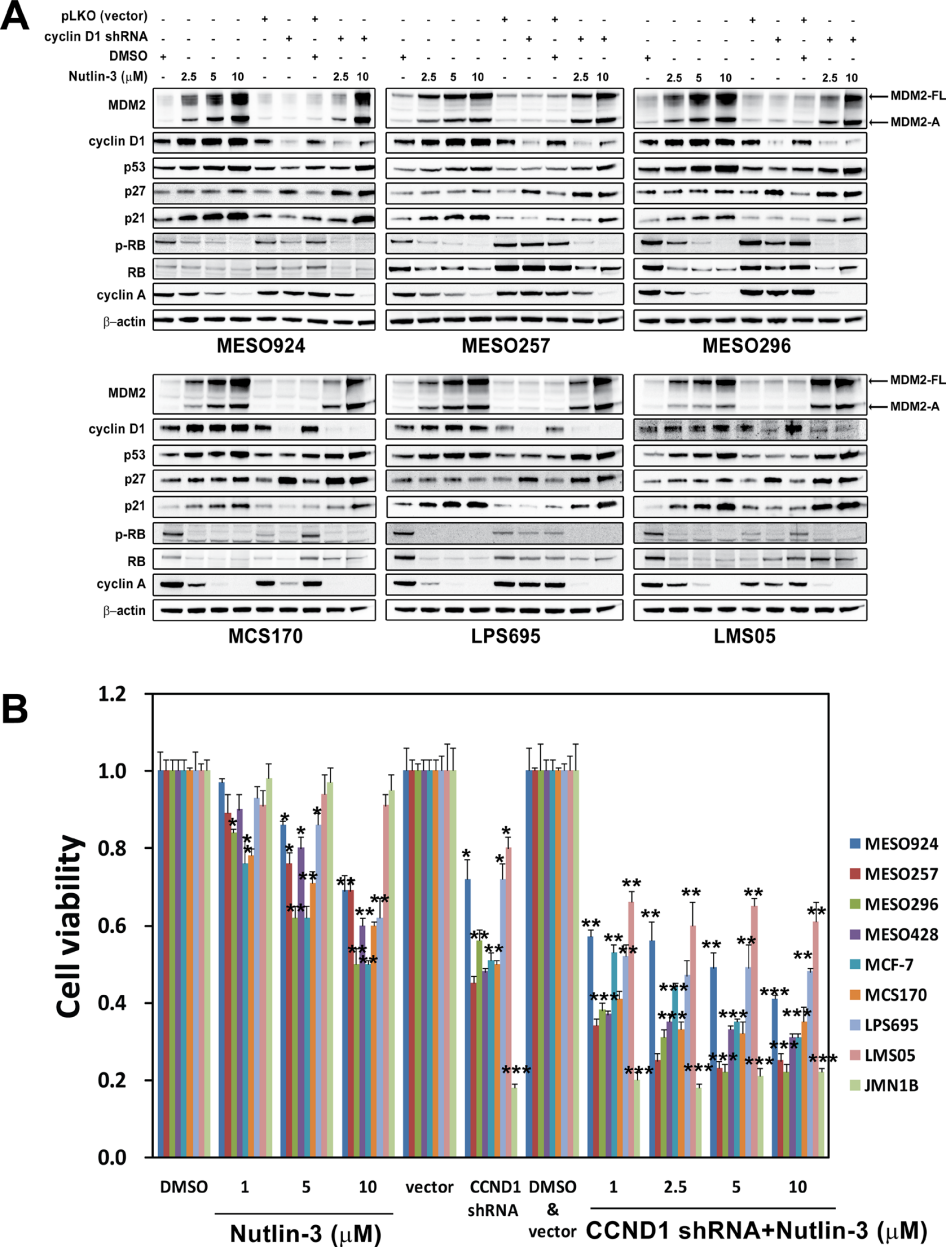
Additive effects were observed through coordinated inhibition of MDM2-p53 interaction and cyclin D1 as demonstrated by immunoblotting (A) and cell viability (B), showing that combination of MDM2 inhibition and cyclin D1 knockdown had greater anti-proliferative effects, compared to either intervention alone in mesothelioma cell lines (MESO924, MESO257, MESO296, MESO428, and JMN1B), a breast cancer cell line (MCF-7), a chondrosarcoma (MCS170), a liposarcoma cell line (LPS695), and a leiomyosarcoma cell line (LMS05) (A) MDM2, cyclin D1, p53, p27, p21, phospho-RB, RB, and cyclin A were evaluated by immunoblotting after treatment with Nutlin-3 for 48 hours and infection with lentiviral *CCND1* shRNA for 72 hours. Actin staining is a loading control. (B) Cell viability evaluated by a Cell-titer Glo^®^ ATP-based luminescence assay in these cell lines, after treatment with Nutlin-3 for 48 hours and infection with lentiviral *CCND1 shRNA* for 72 hours. Data were normalized to the empty vector infections, DMSO, or vector and DMSO, and represent the mean values (± s.d.) from quadruplicate cultures. Statistically significant differences between DMSO and Nutlin-3, empty vector control and target gene shRNAs are presented as *p < 0.05, **p < 0.01, ***p < 0.001.

Due to the observation of cyclin D1 induction after treatment with Nutlin-3, we expected to see additive inhibitory effects on cell viability through coordinated suppression of MDM2-p53 binding and cyclin D1 knockdown or inhibition. *CCND1* gene expression was silenced by lentivirus-delivered shRNAs, and MDM2-p53 binding was inhibited by Nutlin-3 treatment. Cell lines with wild-type p53 (MESO924, MESO257, MESO296, MCS170, LPS695, and LMS05) were evaluated after 3 days of cyclin D1 knockdown and 48 hours of Nutlin-3 treatment. Nutlin-3 treatment alone induced an increase in MDM2, p53, p21, cyclin D1, and cyclin E expression (Figure 3A and Figure S1), and a decrease in phospho-RB, total RB, and cyclin A in a dose-dependent manner. Cyclin D1 knockdown resulted in greater than 80% reduction of cyclin D1 and an increase in p27 and cyclin E expression. The combination of cyclin D1 knockdown and MDM2-p53 inhibition by Nutlin-3 resulted in increased MDM2, p53, p21, p27, and cyclin E expression and reduced phospho-RB, RB, and cyclin A expression to a greater extent than with either intervention alone (Figure 3A and Figure S1).

Additive inhibitory effects on cell viability were obtained through coordinated inhibition of cyclin D1 and MDM2-p53. Cyclin D1 knockdown in wild-type p53 cancer cell lines (MESO257, MESO296, MESO428, MCF-7, MCS170, LPS695, MESO924, and LMS05) and in mutant p53 JMN1B cells resulted in ~45–55% and ~20% inhibition of cell viability at 3 days after cyclin D1 silencing, respectively, compared to the empty vector control (Figure 3B). In addition, cell viability was evaluated after inhibition of cyclin D1 expression by small-molecule inhibitors 4-CPA or CyP (Figure S2), which have been demonstrated to inhibit *CCND1* promoter activity and breast cancer cell growth [20, 21]. Treatment with 50 μM CyP resulted in ~50% reduction in viability at day 3 in MESO924, MESO296, and MCS170. However, viability was minimally suppressed in MESO257 and JMN1B cells after treatment with 50 μM CyP, and in MESO924, MESO257, MESO296, MCS170, and JMN1B after treatment with 100 μM 4-CPA (Figure S2). In wild-type p53 cells (except for LMS05), the most striking reduction in cell viability (30–50%) was seen after treatment with Nutlin-3 (Figure 3B). However, viability was minimally inhibited in mutant p53 JMN1B and wild-type p53 LMS05 cells after treatment with Nutlin-3 (Figure 5B). Combination of *CCND1 shRNA* (day 4) or cyclin D1 inhibition (50 μM CyP or 100 μM 4-CPA for 3 days) and MDM2 inhibition resulted in 60–70% or 40–60% reduction in viability for cell lines with wild-type p53, respectively (Figure 3B and Figure S2). Notably, *CCND1 shRNA* knockdown or drug inhibition resulted in ~80% or 30–50% reduction in viability in JMN1B cells, respectively (Figure 3B and Figure S2).

**Figure 4:**
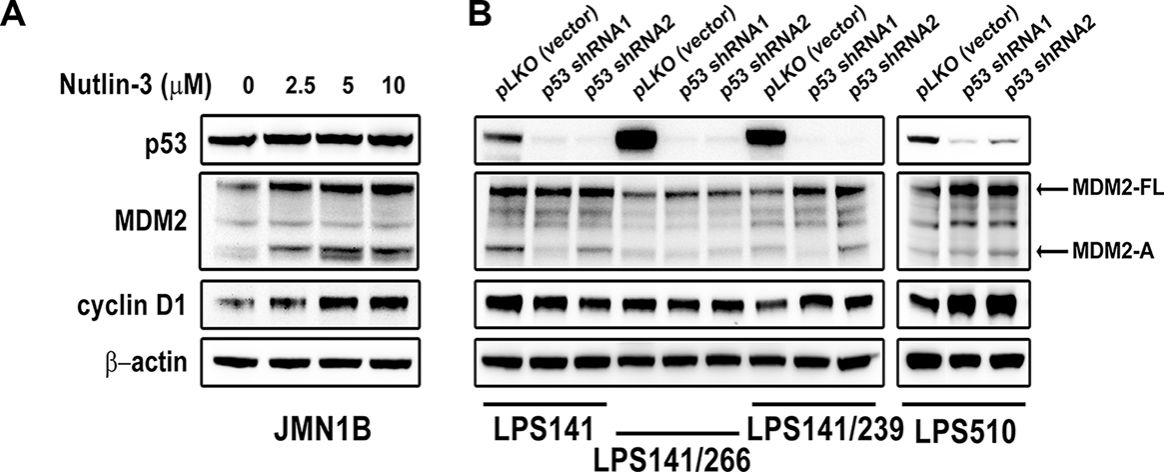
Immunoblotting evaluation of MDM2, p53, and cyclin D1 expression after treatment with Nutlin-3 (2.5, 5, 10 μM) for 48 hours in mutant p53 mesothelioma cell line (JMN1B) (A) and stable p53 knockdowns at 10 days post-infection by lentiviral *TP53* shRNA constructs in liposarcoma cell lines (LPS141, LPS141/239, LPS141/266 and LPS510) (B) β-actin stain is a loading control.

**Figure 5:**
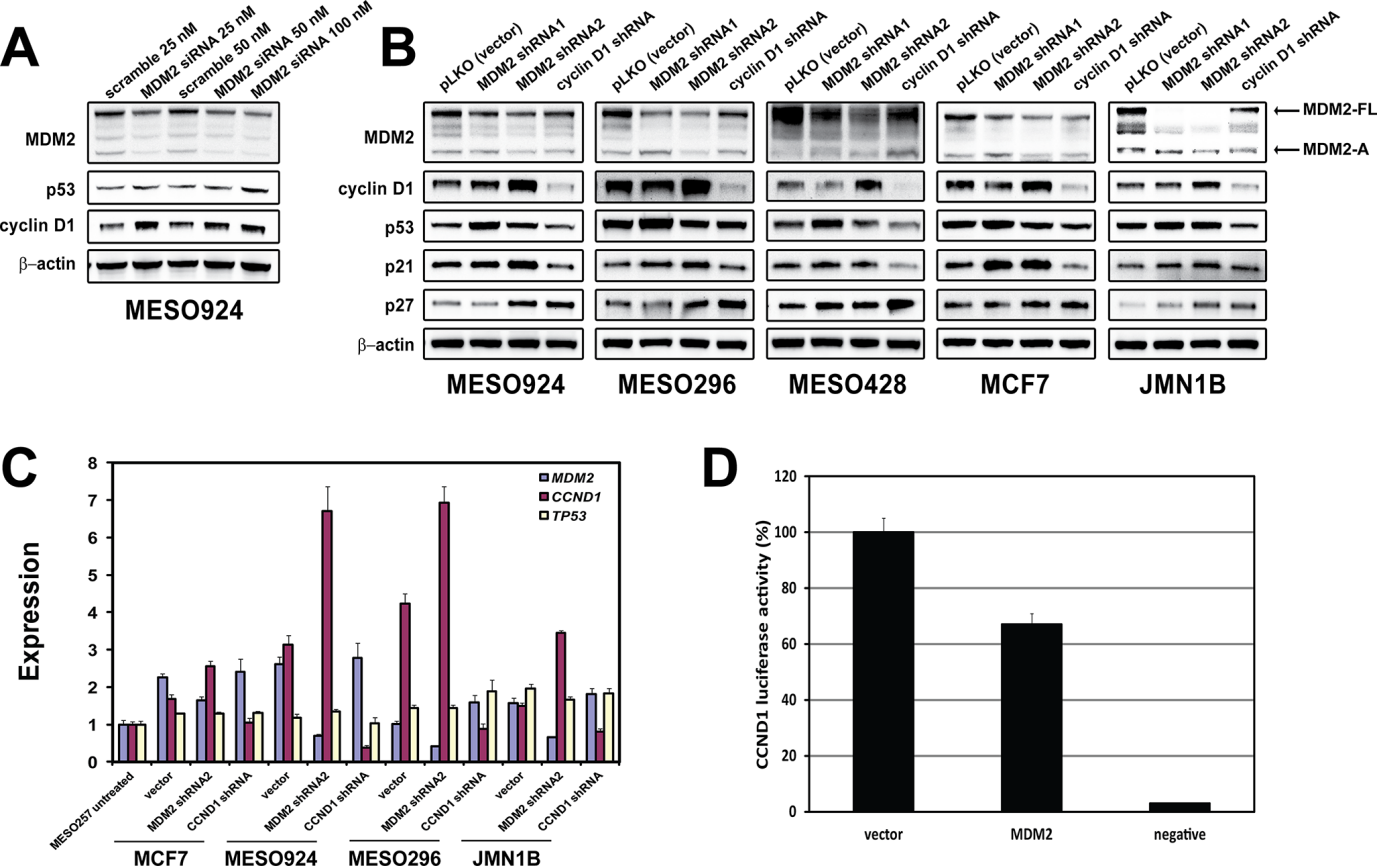
Regulation of cyclin D1 expression by MDM2 (**A**) Immunoblotting evaluation of MDM2, p53, and cyclin D1 expression at 48 hours post-transfection in a mesothelioma cell line (MESO924). (**B**) Expression of MDM2, cyclin D1, p53, p21, and p27 was evaluated by immunoblotting at 96 h post-infection with *MDM2* and *CCND1* shRNAs in four mesothelioma cell lines (MESO924, MESO296, MESO428, and JMN1B) and a breast cancer line (MCF-7). Actin staining is a loading control. (**C**) qRT-PCR shows upregulation of *CCND1* expression in three mesothelioma cell lines (MESO924, MESO296, and JMN1B) at 96 h post-infection with *MDM2* shRNA2, but not *TP53*. The comparative C_t_ (cycle threshold) method was used to determine RNA expression, which was normalized to MESO257 in triplicate assays. (**D**) MDM2 expression inhibits *CCND1* promoter activity: *CCND1* luciferase reporter plasmid pA3−1745CD1LUC (0.25 μg), *Renilla* luciferase reporter plasmid *pTK-RL* (0.005 μg) and *MDM2* or pcDNA3 empty vector (0.25 μg) were cotransfected in 293T cells. Transfected cells were harvested at 48 h, and assessed using a Dual-Luciferase Reporter Assay System. Transfection efficiencies were normalized to the pTK-RL luciferase plasmid, and *CCND1* luciferase activities were normalized to the pcDNA3 vector (100%).

### Correlation of cyclin D1 expression with MDM2, but not p53

We investigated cyclin D1 expression in mutant p53 JMN1B after treatment with Nutlin-3 for 48 hours or in three mutant p53 LPS cell lines (LPS141/266, LPS141/239, and LPS510) and wild-type p53 LPS141 cells after *TP53* gene expression was stably silenced by lentiviral shRNAs. Immunoblotting showed that cyclin D1 induction was accompanied by an increase in MDM2-FL and MDM2-A expression but not p53 in JMN1B after Nutlin-3 treatment (Figure 4A). Silencing of p53 induced MDM2-FL and cyclin D1 expression in LPS141/239 and LPS510, however, p53 knockdown had minimal effect on the expression of MDM2-FL and cyclin D1 in LPS141 and LPS141/266 cells (Figure 4B).

The effect of *MDM2* silencing on cyclin D1 expression in MESO924, MESO296, MESO428, MCF7, and JMN1B was evaluated by immunoblotting after transfection by *MDM2* siRNA or transduction by lentiviral shRNAs (Figure 5A and 5B). We achieved greater than 50% knockdown of MDM2-FL and MDM2-A with siRNA transfection in MESO924. MDM2 knockdown increased cyclin D1 and p53 expression in MESO924 (Figure 5A). Two *MDM2* shRNA constructs showed different effects on cyclin D1 and p53 expression. The *MDM2* shRNA1/2 transductions resulted in greater than ~50% knockdown of MDM2-FL, while the *MDM2* shRNA2 decreased MDM2-A expression, but *MDM2* shRNA1 had minimal effect on MDM2-A expression (Figure 5B). *MDM2* shRNA1 knockdown increased p53 and p21 expression in wild-type p53 cell lines, induced p27 expression in MESO428 and JMN1B, and decreased cyclin D1 expression in MESO428 and MCF7, but had no effect on cyclin D1 expression in MESO924, MESO296, and JMN1B (Figure 5B). *MDM2* shRNA2 induced expression of cyclin D1, p21, and p27 in all cell lines, irrespective of p53 mutation status, but had minimal effect on p53 expression. In addition, cyclin D1 knockdown resulted in induction of p27 (Figure 5B).

Mechanisms of cyclin D1 overexpression were evaluated by qRT-PCR in MCF7, MESO924, MESO296, and JMN1B at 3 days after *MDM2* shRNA2 or *CCND1* shRNA transduction. qRT-PCR demonstrated upregulation of *CCND1* mRNA in the *MDM2*-knockdown cells, with minimal increase in *TP53* mRNA, compared to the empty vector treatment cells (Figure 5C). By contrast, *MDM2* and *TP53* mRNA expression was relatively unchanged after cyclin D1 knockdown in these cell lines (Figure 5C). Furthermore, in dual-luciferase reporter assays, MDM2 inhibited *CCND1* promoter activity by 35% (Figure 5D).

### Cyclin D1 regulation by MDM2 is correlated with expression and ubiquitin E3-ligase activity of MDM2

**Figure 6:**
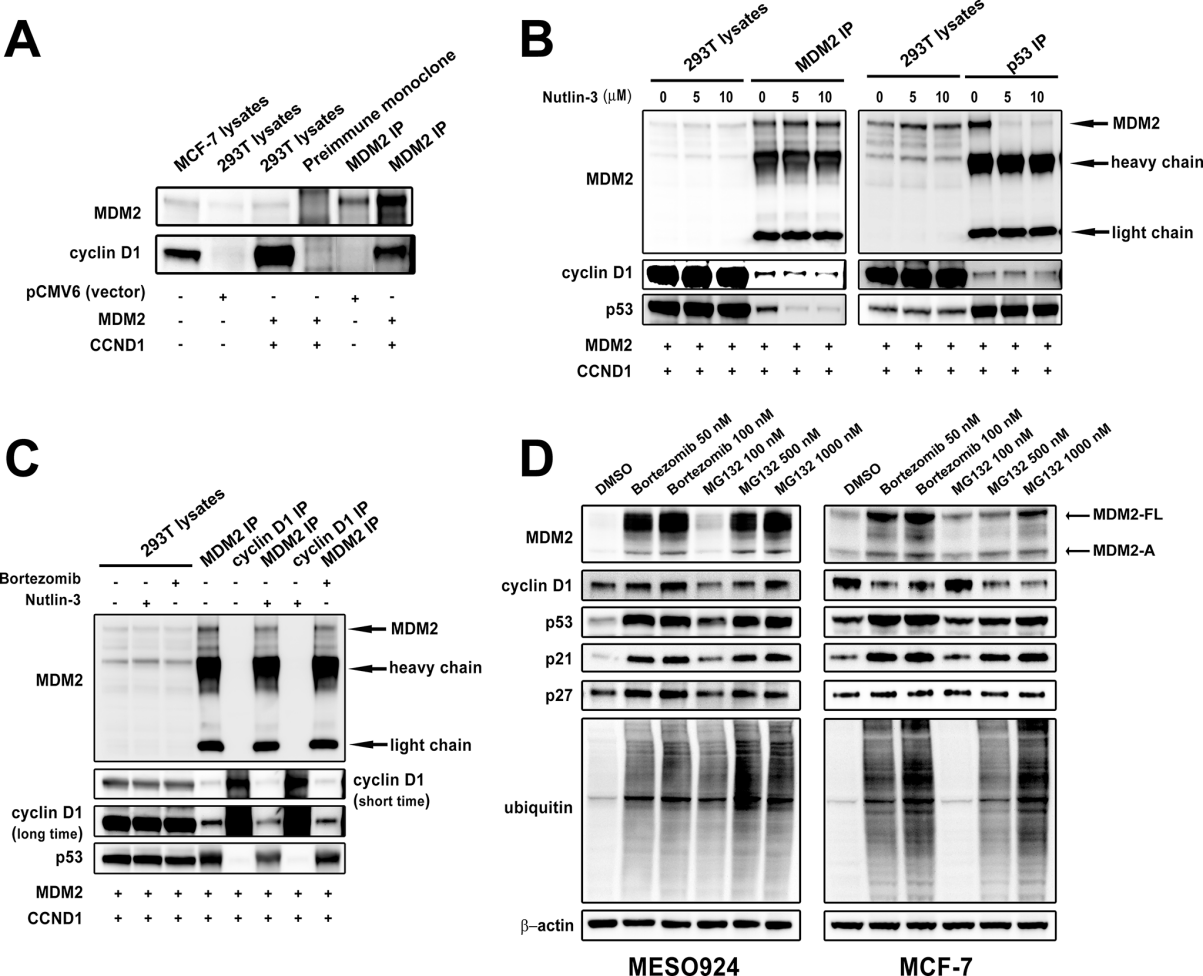
Cyclin D1 regulation by MDM2 depends on expression and ubiquitin E3-ligase activity of MDM2 (**A**) The MDM2-cyclin D1 complex was evaluated in 293T cells at 48 hours post-cotransfection of *MDM2* and *CCND1* by MDM2 immunoprecipitation followed by MDM2 and cyclin D1 immunoblotting. (**B**) Nutlin-3 treatment blocked the interactions of MDM2-cyclin D1 and MDM2-p53. The MDM2-cyclin D1 complex and MDM2-p53 interaction were evaluated in 293T cells at 48 hours post-cotransfection of MDM2 and CCND1 and after treatment with Nutlin-3 (5 and 10 μM) for 24 hours by MDM2 or p53 immunoprecipitation, followed by MDM2, cyclin D1 and p53 immunoblotting. (**C**) The interaction of MDM2-cyclin D1 is correlated with ubiquitin E3-ligase activity of MDM2. The MDM2-cyclin D1 complex was evaluated in 293T cells at 48 hours post-cotransfection of MDM2 and CCND1 and after treatment with Nutlin-3 (10 μM) and bortezomib (100 nM) for 24 hours by MDM2 or cyclin D1 immunoprecipitation, followed by MDM2, cyclin D1 and p53 immunoblotting. (**D**) Immunoblotting evaluation of MDM2, cyclin D1, p53, p21, p27, and ubiquitin expression after treatment with bortezomib (50 and 100 nM) and MG132 (100, 500, and 1000 nM) for 24 hours in MESO924 and MCF-7. β-actin stain is a loading control.

To evaluate the interaction of MDM2 and cyclin D1, we cotransfected 293T cells with *CCND1* and *MDM2* expression constructs and performed MDM2 immunoprecipitations (co-IP) and MDM2 and cyclin D1 immunoblotting (Figure 6A). MDM2 co-IP revealed a dominant cyclin D1 34 kDa band. By contrast, preimmune serum co-IP did not show any bands at the same position (Figure 6A). We next evaluated whether Nutlin-3 treatment affected the binding of MDM2 and cyclin D1 by MDM2 and p53 co-IPs after Nutlin-3 treatment followed by MDM2, cyclin D1, and p53 immunoblotting (Figure 6B). As compared to the DMSO control treatment, Nutlin-3 treatment decreased the cyclin D1 and p53 bands in MDM2 co-IPs, and the MDM2 bands in p53 co-IPs (Figure 6B). Furthermore, MDM2 co-IPs showed a decrease in MDM2:cyclin D1 interaction after Nutlin-3 treatment, and bortezomib treatment had little effect on this interaction (Figure 6C). Finally, immunoblotting evaluated cyclin D1 expression in MESO924 and MCF7 after treatment with bortezomib and MG132 for 24 hours, which block the degradation of ubiquitin-tagged proteins (Figure 6D). Bortezomib and MG132 treatment resulted in cyclin D1 accumulation and an increase in ubiquitination, accompanied by accumulation of MDM2, p53, p21, and p27 in MESO924. However, bortezomib and MG132 treatment decreased cyclin D1 expression while causing an increase in ubiquitination and expression of MDM2, p53, and p21 in MCF7 (Figure 6D).

## DISCUSSION

MDM2 plays a central role among multiple mechanisms responsible for the regulation of p53 activity [22]. MDM2 inhibits p53 by binding to the N-terminal domain of p53 and masking p53's access to transcriptional machinery, and by ubiquitinating p53, marking it for proteasomal degradation. Therefore, inhibition of MDM2-p53 binding is an attractive target for restoration of p53 activity in tumor cells, and small-molecule MDM2 inhibitors have shown promise in previous preclinical studies [23, 24] and in the present work (Figure 3B and Figure S2).

Previous studies have suggested that the *MDM2*-amplified subset of wild-type p53 tumors might be particularly sensitive to MDM2 inhibition [12]. Recently, the relative expression of MDM2, MDM2-B, MDMX (MDM4), and MDMX-S has been found to predict heterogeneous responses of Nutlin-3 in cancer cells [16]. The present data showed that Nutlin-3 treatment resulted in decreased phosphorylation and expression of RB in tumor cells, accompanied by an increase in MDM2-FL and MDM2-A expression (Figure 3A). These findings are in keeping with previous evidence for regulation of RB activation and expression by MDM2 and MDMX [25, 26]. Additionally, inhibition of the MDM2-p53 interaction induced cyclin D1 and cyclin E expression (Figure 2, Figure 3A, and Figure S1), which is consistent with published data showing that expression of MDM2-A resulted in induction of cyclin D1 and cyclin E in Hodgkin's lymphoma cells [17]. We investigated whether the induction of cyclin D1 attenuated the response to Nutlin-3 in these cancer cells. Additive effects were obtained through simultaneous MDM2 inhibition and cyclin D1 shRNA knockdown/drug inhibition (Figure 3B and Figure S2), as shown by a decrease in cyclin A expression and reduction of cell viability. These findings highlight that combination inhibition of MDM2-p53 interaction and cyclin D1 expression warrants clinical evaluation as a therapeutic strategy in wild-type p53 cancers.

To determine whether the induction of cyclin D1 after Nutlin-3 treatment was correlated with p53 mutational status, we evaluated MDM2, p53, and cyclin D1 expression in a p53-mutant cell line (JMN1B) after Nutlin-3 treatment. Nutlin-3 treatment resulted in upregulation of cyclin D1 accompanied by the increase of MDM2-FL and MDM2-A, with no effect on mutant p53 expression (Figure 4A). p53 knockdown by shRNAs induced expression cyclin D1 and MDM2-FL in mutant p53 LPS510 and LPS141/239, but resulted in no changes in expression in mutant p53 LPS141/266 and wild-type p53 LPS141 cell lines (Figure 4B). These results indicate that cyclin D1 expression might be inhibited by MDM2 but not p53, and there is no consistent response to p53 knockdown in the cell lines tested.

To demonstrate cyclin D1 suppression by MDM2, MDM2 was silenced by siRNA and lentiviral shRNAs (Figure 5A and 5B). Knockdown of MDM2-FL and MDM2-A by siRNA induced cyclin D1 expression (Figure 5A). *MDM2* shRNA1 knockdown induced p53 expression without changing cyclin D1 expression. *MDM2* shRNA2 knockdown resulted in upregulation of cyclin D1, without changing p53 expression (Figure 5B). Although both *MDM2* shRNA constructs inhibited MDM2-FL expression, only shRNA2 effectively suppressed MDM2-A expression (Figure 5B). qRT-PCR showed that *MDM2* shRNA2 knockdown increased *CCND1* transcript levels, with minimal effect on p53 levels (Figure 5C). In addition, MDM2 inhibited *CCND1* promoter activity, as shown by luciferase reporter assays (Figure 5D). These findings indicate that MDM2-A may play a role in regulating cyclin D1, and this is likely a p53-independent mechanism. This finding is in line with increasing evidence that MDM2 promotes tumorigenesis in a p53-independent manner in cells lacking p53, in cells with mutant p53, and in cells expressing MDM2 splice variants lacking a p53-binding domain but possessing transforming activity [11, 27].

The *MDM2* shRNA2 sequence binds the sequence coding for the COOH-terminal domain of MDM2 containing E3 ubiquitin ligase activity. After *MDM2* shRNA2 transduction, MDM2-FL and MDM2-A silencing was accompanied by cyclin D1 induction (Figure 5B). Thus, we hypothesize that cyclin D1 expression depends on the ubiquitin E3-ligase activity of MDM2. We demonstrated by immunoprecipitations that MDM2 binds with cyclin D1 after overexpression of MDM2 and cyclin D1 in 293T cells (Figure 6A). Nutlin-3 treatment dramatically decreased the binding of MDM2 and p53, and partially attenuated cyclin D1 binding by MDM2 (Figure 6B). Furthermore, bortezomib treatment resulted in minimal inhibition of the binding of MDM2 and cyclin D1/p53 in these cells, but inhibition of the MDM2-p53 interaction by Nutlin-3 attenuated the interaction of MDM2 and cyclin D1/p53 (Figure 6C). Inhibition of proteasome activity by bortezomib and MG132 increased cyclin D1 expression in MESO924, but decreased cyclin D1 expression in MCF-7 (Figure 6D). These findings indicate that cyclin D1 expression is correlated with expression and ubiquitin E3-ligase activity of MDM2. In the present study, MDM2 knockdown resulted in downregulation (*MDM2* shRNA1) and upregulation (*MDM2* shRNA2 and *MDM2* siRNA) of cyclin D1 (Figure 5B), suggesting that complicated mechanisms may be involved for the different isoform expression of MDM2.

In summary, we have shown that small-molecule inhibition of MDM2-p53 interaction induces cyclin D1 expression, and that regulation of cyclin D1 by MDM2 involves different isoforms. Cyclin D1 expression is correlated with the expression and ubiquitin E3-ligase activity of MDM2. Finally, a multipoint targeted attack on MDM2-p53 interaction and cyclin D1 could potentially maximize the treatment effect of MDM2-p53 inhibitors by blocking the compensatory upregulation of cyclin D1.

## MATERIALS AND METHODS

### Antibodies and reagents

Monoclonal antibodies to p53, cyclin A, cyclin D1, cyclin E, ubiquitin, preimmune serum, and *MDM2* siRNA (sc-29394) were from Santa Cruz Biotechnology (Santa Cruz, CA). Monoclonal antibody to RB and polyclonal antibody to phospho-RB (Ser807/811) were from Cell Signaling Technology (Beverly, MA). Monoclonal antibody to p27 was from BD Transduction Laboratories (San Jose, CA). Antibodies to MDM2 and p21, and Protein A- and Protein G-sepharose beads were purchased from Invitrogen Laboratories (Invitrogen Life Technologies, Carlsbad, CA). Polybrene, puromycin, *CCND1* shRNA constructs, 2-Cyclopenten-1-one (CyP) and 4-chlorophenylacetate (4-CPA), and antibody to β-actin were from Sigma-Aldrich (St, Louis, MO). Bortezomib and MG132 were obtained from LC Labs (Woburn, MA) and EMD Millipore (Billerica, MA), respectively. These inhibitors were reconstituted in DMSO. Lentiviral shRNA constructs were from The RNAi Consortium (TRC, Cambridge, MA, USA), and included *MDM2*: CTTTGGTAGTGGAATAGTGAA (shRNA1); CTCAGCCATCAACTTCTAGTA (shRNA2), and *TP53*: CTTCGACTATCTCAAACTCCT (shRNA1), CAAGGTACTTCGATGATGAAT (shRNA2).

### Cancer cell lines

Mesothelioma cell lines were established from epithelial-type mesotheliomas (MESO924, MESO257 and JMN1B) [28–30], or mixed histology mesothelioma (MESO296), or spindle-cell mesothelioma (MESO428) [28, 29]. MESO924, MESO257, MESO296, and MESO428 have wild-type p53, but JMN1B contains a p53 point mutation (G245S). All cell lines were validated by unique clonal cytogenetic aberrations within 10 passages of the present studies (MESO924, MESO257, MESO296, and MESO428 were validated by comparison with the corresponding surgical specimens, and JMN1B was validated by comparison with published cytogenetic aberrations). LPS141, LPS141/266, LPS141/239, LPS695, and LPS510 were developed in the Department of Pathology at Brigham and Women's Hospital. The LPS141 cell line was developed from a dedifferentiated liposarcoma (DDLPS) with heterologous osteosarcoma, arising in a patient with a history of recurrent well-differentiated liposarcoma (WDLPS) [31]. LPS141/266 and LPS141/239 are isogenic sublines of LPS141 which retain the parental *MDM2* and *CDK4* genomic amplification but have acquired p53 point mutations (G266R and N239D, respectively). The LPS695 cell line harboring wild-type p53 was developed from a DDLPS in a patient with a history of WDLPS [31]. LPS510 contains *MDM2* and *CDK4* genomic amplification, and a p53 point mutation (H179R). LMS05 and MCS170 were established in Dr. Jonathan Fletcher's laboratory from a primary thigh leiomyosarcoma and chondrosarcoma, respectively. GIST882 and GIST430 are *KIT*-mutant human GIST cell lines established from untreated GIST (GIST882) or from GISTs progressing on imatinib therapy (GIST430) [32]. Ovarian cancer cell lines (SKOV3, OVCA429, and ES2) are kind gifts from Dr. Ross Berkowitz in the Laboratory of Gynecologic Oncology at Brigham and Women's Hospital and Harvard Medical School. Breast cancer cell line MCF-7 and non-small cell lung cancer cell lines (PC-9 and A549) were from the cell library of the Chinese Academy of Sciences in Shanghai.

### Protein lysate preparations and immunoblotting

Immunoblotting was performed after 48 or 24 hours of treatment with Nutlin-3 or Bortezomib/MG132, respectively, or after 4 days post-transfection with MDM2 siRNA, and after 3 or 10 days post-infection with *MDM2/CCND1* or *p53* shRNAs. Whole cell lysates were prepared using lysis buffer (1% NP-40, 50 mM Tris-HCl pH 8.0, 100 mM sodium fluoride, 30 mM sodium pyrophosphate, 2 mM sodium molybdate, 5 mM EDTA, and 2 mM sodium orthovanadate) containing protease inhibitors (10 μg/mL aprotinin, 10 μg/mL leupeptin, and 1 mM phenylmethylsulfonyl fluoride). Lysate protein concentrations were determined using a Bio-Rad protein assay (Bio-Rad Laboratories Hercules, CA, USA). Electrophoresis and western blotting were performed as described previously [33]. The hybridization signals were detected by chemiluminescence (Immobilon^™^ Western, Millipore Corporation, MA) and captured using a GE FUJI ImageQuant LAS4000 chemiluminescence imaging system.

### Immunoprecipitation

Sepharose-protein G beads linked to mouse monoclonal antibodies and sepharose-protein A beads linked to rabbit polyclonal antibody were used for immunoprecipitation. One mg of protein lysate (500 μL) was preadsorbed for 30 min at 4°C using 20μl of protein G/A beads. 3 μg of primary mouse antibodies against MDM2, p53, and primary rabbit antibodies against cyclin D1 were rocked with the lysates for 2 hours at 4°C. Then, 20 μL of sepharose-protein G/A beads was added and rocked overnight at 4°C, then centrifuged at 10,000 rpm for 2 min at 4°C, after which the sepharose beads were washed 3 times (25 min each) with 750 μL of IP buffer and once with 750μL 10 mM Tris-Cl buffer (pH7.6). Loading buffer (20 μL) was added to the beads and boiled for 5 min at 95°C.

### Transfection

Transfection of *MDM2* siRNA in MESO924, and cotransfection of *CCND1* and *MDM2* expression vectors in 293T cell was carried out according to the manufacturer's instructions, using lipofectamine and PLUS reagent (Invitrogen Life Technologies, Carlsbad, CA). Briefly, siRNA or DNA was incubated with PLUS reagent in 100 μl of serum-free medium for 15 min at room temperature (RT). Lipofectamine was diluted with 100 μl of serum-free medium and added to the DNA/RNA and PLUS complexes, and then incubated another 15 min at room temperature. Finally, DNA/RNA-PLUS-lipofectamine complexes were added to 60% confluent cultures of MESO924 or 293T cells in serum-free media in six-well plates. DNA/RNA-PLUS-lipofectamine complexes in serum-free medium were completely replaced with serum-containing regular media after 3 h incubation. Cells were lysed for western blot analysis at 48 hours post-transfection.

### Preparation of shRNA lentiviruses

Lentiviruses were produced by cotransfecting pLKO.1puro plasmids containing *MDM2*, *p53* or *CCND1* shRNAs, and helper virus packaging plasmids pCMVΔR8.91 and pMD.G (at a 10:10:1 ratio) into 293T cells. Transfections were carried out using lipofectamine and PLUS reagent (Invitrogen Life Technologies, Carlsbad, CA). Lentiviruses were harvested at 24, 36, 48, and 60 hours post-transfection. Virus was frozen at −80°C in appropriately sized aliquots for infection. Well-validated shRNAs were used for MDM2, p53, and cyclin D1 knockdowns.

### Cell culture and virus infection

Mesothelioma cell lines (MESO924, MESO257, MESO296, MESO428, and JMN1B), LPS cell lines (LPS141, LPS695, LPS141/239, LPS141/266, and LPS510), MCF-7, and MCS170 were maintained in RPMI 1640 with 10% fetal bovine serum (FBS) supplemented with penicillin/streptomycin and 1% (v/v) L-glutamine. Cells were seeded in six-well plates and lentiviral *MDM2*, *CCND1*, or *p53* shRNA infections were carried out in the presence of 8 μg/mL polybrene. All lentiviral experiments were performed in duplicate. Following transduction, LPS141, LPS141/239, LPS141/266, and LPS510 cells were selected for stable expression of the *p53* shRNAs using 2 μg/ml puromycin. Cells were lysed for western blotting at 3 days or 10 days post-infection.

### Cell proliferation and apoptosis assays

Cell lines were plated at 3,000–5,000 cells/well in a 96-well flat-bottomed plate (Falcon, Lincoln NJ) and cultured for 24 hours before treatment with different inhibitors, which included Nutlin-3 (1, 2.5, 5, and 10 μM), CyP (50 μM), 4-CPA (100 μM), and *CCND1* shRNA. Cell viability was determined after treatment with inhibitors or shRNA for 48 or 72 hours, respectively, using the CellTiter-Glo luminescent assay (Promega, Madison, WI) and measured using a Veritas^™^ Microplate Luminometer (Turner Biosystems, Sunnyvale, CA). The data were normalized to the control group (empty vector or DMSO). The IC_50_ value was defined as the concentration that causes 50% growth inhibition. IC_50_ values were calculated using a sigmoidal curve fit with GraphPad Prism Software (GraphPad Software, Inc., La Jolla, CA). All experimental points were set up in four replicate wells and independently performed in duplicate.

### RNA preparation and qRT-PCR

*MDM2*, *CCND1*, and *TP53* RNA expression was evaluated by qRT-PCR after treatment with shRNA for 72 hours in MCF-7, MESO924, MESO296, and JMN1B. Total RNA was prepared using Trizol reagent (Invitrogen, Carlsbad, CA). RT-PCR was performed using 1 μg RNA, with the Bio-Rad iScript^™^ cDNA synthesis Kit (Bio-Rad Laboratories, Hercules, CA). qPCR was performed with iQ SYBR green supermix (Bio-Rad) in a reaction volume of 25 μl, using a MyiQ single-color real-time PCR detection system (Bio-Rad). Reactions contained 1 μl cDNA, 400 nM of each primer, and 12.5 μl iQ SYBR green supermix. After 10 min at 95^°^C, each of the 40 PCR cycles consisted of denaturation for 10 seconds at 95^°^C and hybridization of primers and SYBR green as well as DNA synthesis for 1 minute at 60^°^C. The qRT-PCR assays for *CCND1*, *MDM2*, and *TP53* were performed using the following primers: *CCND1* (NM_053056) sense: 5′-CCGTCCATGCGGAAGATC-3′ and anti-sense: 5′-GAAGACCTCCTCCTCGCACT-3′ [34]; *MDM2* (NM_002392) sense: 5′-ACCTCACAGATT CCAGCTTCG-3′ and anti-sense: 5′-TTTCATAGTATA AGTGTCTTTTT-3′ [35]; *TP53* (NM_000546) sense: 5′-TAACAGTTCCTGCATGGGCGGC-3′ and anti-sense: 5′-AGGACAGGCACAAACACGCACC-3′ [36]. As controls, *GAPDH* was amplified using the following primers: *GAPDH* (NM_002046) sense: 5′-GAAGGTGA AGGTCGGAGTCAAC-3′ and anti-sense: 5′-TGGAAGAT GGTGATGGGATTTC-3′. All primers were obtained from Invitrogen (Invitrogen Life Technologies, Carlsbad, CA). The comparative C_t_ (cycle threshold) method was used to determine RNA expression fold differences in GIST cell lines. The data points (run in triplicate assays) were normalized to *GAPDH*.

### Dual luciferase assay

293T cells were transfected using lipofectamine and PLUS reagent. For each experiment, *CCND1* firefly luciferase reporter plasmid pA3−1745CD1LUC (0.25 μg), *Renilla* luciferase reporter plasmid *pTK-RL* (0.005 μg) and *MDM2* or pcDNA3 (0.25 μg) were cotransfected. Transfection of the pcDNA3 empty vector alone served as a negative control in the reporter assays. The relative luciferase activity was calculated based on the amount of luminescence produced by *Renilla* luciferase for each reaction. Transfected cells were harvested 48 hours post-transfection, and results were obtained by using the Dual-Luciferase Reporter Assay System, according to the manufacturer's instructions (Promega). At least three independent experiments were performed. Transfection efficiencies were normalized to the *pTK-RL* luciferase plasmid. *CCND1* luciferase activities were normalized with the pcDNA3 vector.

### Statistical analysis

Student's *t*-tests were performed on data from cells treated with control DMSO or inhibitors, as well as cells treated with empty vector or *CCND1* shRNA. Statistically significant differences between untreated control and treatment were defined as *P < 0.05, **P < 0.01 and ***P < 0.001.

## SUPPLEMENTARY MATERIAL FIGURES


