# Supplementary material for: Downregulation of cyclin D1 sensitizes cancer cells to MDM2 antagonist Nutlin-3
Source: Oncotarget. 2016 Apr 26;7(22):32652–63. doi: 10.18632/oncotarget.8999 (PMC5078041; doi:10.18632/oncotarget.8999)
Supplement: Supplementary file 1 [file oncotarget-07-32652-s001.pdf]

Downregulation of cyclin D1 sensitizes cancer cells to MDM2 antagonist Nutlin-3

Supplementary Materials

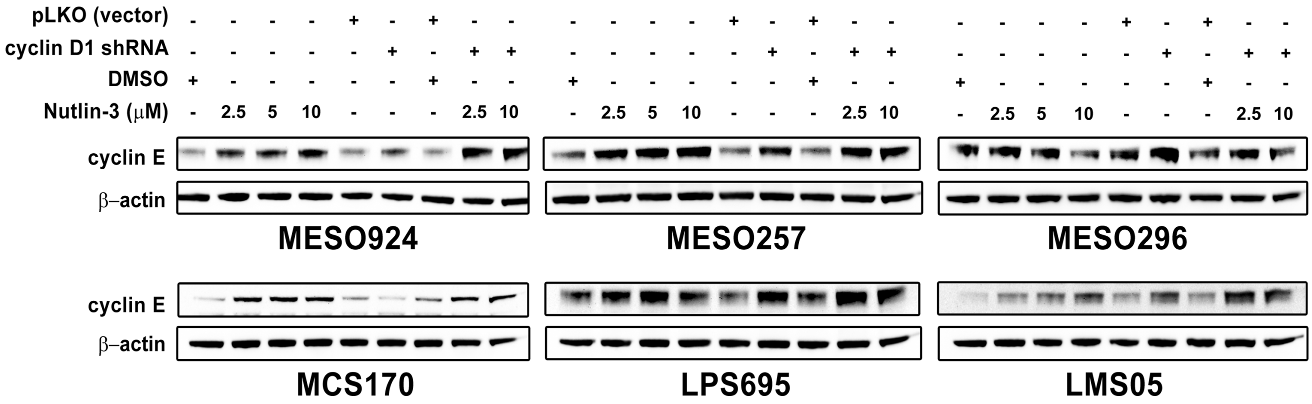

Supplementary Figure S1: Cyclin E was evaluated by immunoblotting after treatment with Nutlin-3 for 48 hours and infection with lentiviral CCND1 shRNA for 72 hours. Actin staining is a loading control.

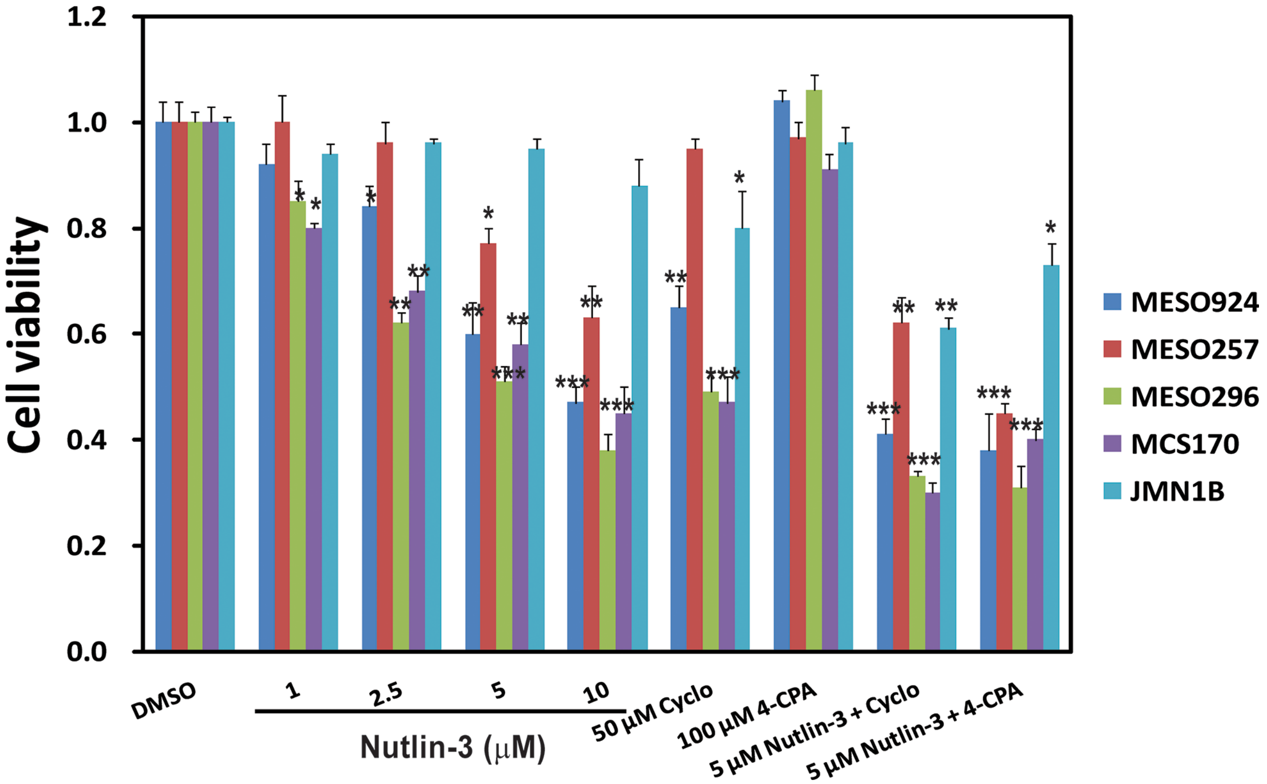

Supplementary Figure S2: Additive effects were observed through coordinated inhibition of MDM2-p53 interaction and cyclin D1 as demonstrated by cell viability, showing that combination inhibition of MDM2 and cyclin D1 has greater anti-proliferative effects, compared to either intervention alone in mesothelioma cell lines (MESO924, MESO257, MESO296, and JMN1B), and chondrosarcoma cell line (MCS170). Cell viability was evaluated by a Cell-titer Glo® ATP-based luminescence assay in these cell lines, after treatment with Nutlin-3 and CyP (50 μM) or 4-CPA (100 μM) for 72 hours. Data were normalized to DMSO, and represent the mean values (± s.d.) from quadruplicate cultures. Statistically significant differences between DMSO control and drug treatment are presented as *$p < 0.05$, **$p < 0.01$, ***$p < 0.001$.
